# The role of ligands in coinage-metal nanoparticles for electronics

**DOI:** 10.3762/bjnano.8.263

**Published:** 2017-12-07

**Authors:** Ioannis Kanelidis, Tobias Kraus

**Affiliations:** 1 INM – Leibniz Institute for New Materials, Campus D2 2, 66123 Saarbrücken, Germany; 2 Department of Chemistry, Saarland University, 66123 Saarbrücken, Germany

**Keywords:** conductivity, inks, layers, ligands, nanoparticles

## Abstract

Coinage-metal nanoparticles are key components of many printable electronic inks. They can be combined with polymers to form conductive composites and have been used as the basis of molecular electronic devices. This review summarizes the multidimensional role of surface ligands that cover their metal cores. Ligands not only passivate crystal facets and determine growth rates and shapes; they also affect size and colloidal stability. Particle shapes can be tuned via the ligand choice while ligand length, size, ω-functionalities, and chemical nature influence shelf-life and stability of nanoparticles in dispersions. When particles are deposited, ligands affect the electrical properties of the resulting film, the morphology of particle films, and the nature of the interfaces. The effects of the ligands on sintering, cross-linking, and self-assembly of particles in electronic materials are discussed.

## Introduction

**Figure 1:**
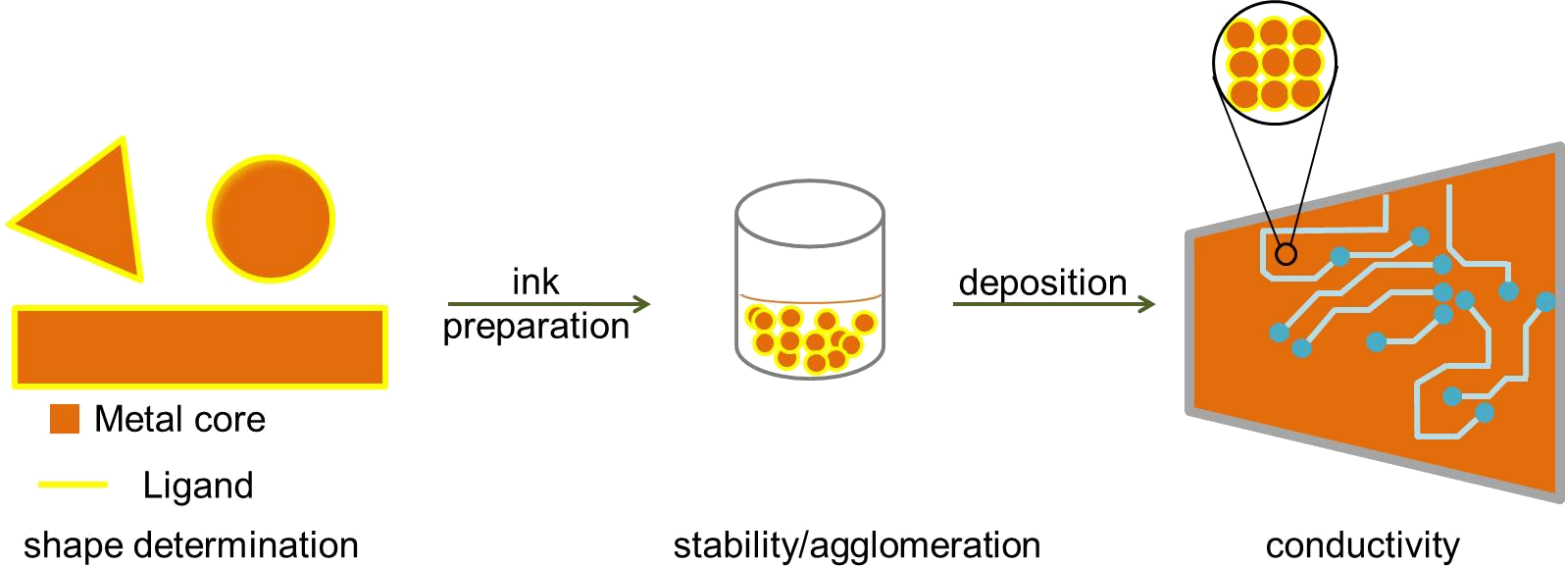
Ligands determine the shape of coinage-metal nanoparticles, affect the stability of their colloids and inks containing them, and the structure and properties of films deposited from such inks.

Nanomaterials with characteristic lengths in the range of 1 to 100 nm, for example in diameter, grain size, or layer thickness, are interesting components for electronic materials [1]. Geometry-directing chemical syntheses allow their preparation with controlled shapes [2]. Coinage-metal nanostructures with a variety of shapes have been created, including spheres [3–5], rods [6–7], wires [8–9], belts [10–11], plates [12–13], and cubes [14]. Synthetic approaches include electrochemical deposition [15], surfactant addition [16], hydrothermal reduction [17], soft templates in solution [6], polymer-mediated synthesis [18–20], thermal decomposition [21], gamma, and electron beam irradiation [22–23], vapor phase deposition [24] and in situ synthesis through inkjet printing [25]. Such metal nanostructures are useful in inks for printed electronics (Figure 1). They are small enough not to limit resolution, they fit through the small nozzles of inkjet printers, they sediment only slowly, and they have lower melting temperatures than the bulk. Many of them carry ligand molecules that need to be considered when using them for electronics. This review is devoted to the role of the ligands.

Surface ligands are employed during synthesis to determine geometry [2,8], to control colloidal stability [5,26–28], to expand the solubility range through end-functionalization [29], or to influence the electro-optical features [26,30] of the resulting nanoparticles (NPs). They can act as cross-linkers and facilitate self-assembly when the particles are applied to solid substrates [31]. Ligands that stabilize nanoparticles in suspension are used for the preparation of stable metal-based inks with increased shelf life. They are essential in order to achieve the high metal loadings required in many printing processes [32–33]. Certain ligands mediate the formation of nanoparticles directly in an ink precursor, thus unifying nanoparticle and ink preparation [25].

The interparticle spacing induced by the ligands helps to stabilize the dispersions, but it also causes insulating barriers after deposition, prevents metallic contact in the deposited layers, and leads to low electrical conductivity [30,34]. Layers prepared from concentrated nanosized coinage-metal inks usually only become electrically conductive after a post-deposition sintering that removes ligands and fuses the metal nanoparticles. Sintering has been achieved by thermal decomposition of the ligands [35–36], light [37–38], plasma treatment [39], microwaves [40], electrical voltage applied to the film [41], chemical agents [42], or ageing [43]. Thermal decomposition, the conventional method for welding metal nanoparticles together, requires temperatures that depend on the particle size and binding strength of the ligand [44]. For example, short alkanethiols allowed for the sintering of gold nanoparticles (Au NPs) at temperatures smaller than 150 °C [45], far below the typical gold sintering temperatures of 200–350 °C [35–36]. It is possible to use ligands that do not require high sintering temperatures for temperature-sensitive substrates [46].

Layers of metal-based inks have been used to fabricate conductive electrodes, thin-film transistors, light emitting diodes, and solar cells [32]. Ultrathin films [47], arrays of interconnected rings [48], or patterned grids [49] have been fabricated as transparent conductive electrodes. Inks of metal nanoparticles are also good candidates to print electrodes for organic field-effect transistors [50]. Magdassi et al. demonstrated the inkject printing of silver nanowires (Ag NWs) onto plastic substrates to create highly conductive patterns for an electroluminescent device [42]. Yu et al. successfully used silver nanoparticle (Ag NP) inks to prepare grids for polymer solar cells by inkjet or flexographic printing [51].

Charge transport in spaced metal nanoparticle layers is strongly affected by ligands [52]. Typical conduction mechanisms within such films are tunneling or electron hopping [26,53]. An exponential dependence of the tunneling-dominated film conductivity on the number of saturated carbon–carbon bonds in the ligands has been reported [54–55]. The chemical nature of dithiols or dithiocarbamate derivatives interconnecting nanoparticles influenced film conductivities in a study by Wessels and co-workers: When the authors replaced a benzene ring in the ligand with a cyclohexane ring, the film conductivity dropped. Conjugation in the benzene ligands and their good contact to the Au NPs allowed the electron wave functions between neighboring nanoparticles to overlap and electron transport was described by thermally activated tunneling between the particles. Locating the Fermi level of the metal in the HOMO–LUMO gap of the ligand shell provides a pathway for electrons to pass along [56]. In electron hopping charge carriers are activated thermally and the probability of hopping depends on the distance of the nearest unoccupied electronic state in the conduction band [53,57]. Murray et al*.* showed that electron hopping conductivities in films of gold nanoclusters stabilized by arene thiolates increased when decreasing the number of saturated units [58]. A different study found that π–π-stacking of phenyl groups in films of phenylethylthiolate-stabilized gold nanoparticles determined the interparticle separation and regulated charge transfer. The short ligand chain and the aromatic moieties facilitate charge transfer with the conductivities exhibiting a clear Arrhenius behavior implying charge transfer driven by thermal activation in an electron hopping mechanism. Maximal conductivity was reached when the aromatic groups of ligands of adjacent particles were stacked [59].

Whatever the local conduction mechanism may be, macroscopic conductivity requires at least one continuous path of metal particles between the electrodes. Ligands influence the spatial orientation of the nanoparticles during drying or when forming a composite in a polymer matrix, and this distribution of the NPs affects the percolation threshold, the critical volume fraction of particles required to provide metallic behavior in layers [60–61]. Surface ligands have been tailored to provide interactions that foster organization into structures with sufficient connectivity and provide conductive pathways throughout films prepared from metal colloidal solutions [53,62].

Ligands affect at different stages and through different mechanisms the properties of electronic materials based on coinage metal particles. This review discusses the role of ligands during particle synthesis, in the particle-containing ink, and during deposition, where they may affect self-assembly, cross-linking, or sintering. The effects of ligands at all stages have implications on the electrical properties of the final particle-based materials. Finally, we review first reports on ligands in electrically conductive composites of polymers and metal nanoparticles.

## Review

### 1 Ligands in the synthesis of metal particles

The shapes of coinage-metal nanoparticles during liquid-phase synthesis can be controlled through the introduction of capping ligands in the reaction mixture. Certain ligands determine the final shape of a nanocrystal depending on their binding affinity on different facets. They cover the nuclei as they form from the molecular precursor and prevent their aggregation [63]. Once the nuclei have grown into structurally well-defined seeds, the ligands can lead to preferential capping, hindering or promoting the growth of particular facets [2]. Their structure-directing action is due to a combination of thermodynamic driving forces (energy differences in facets) and transport modulation, where ligand coverage adjusts diffusion of monomers to the facets [2]. Some ligands also form micelles that can act as templates during the initial nucleation processes of particle synthesis; this is probably the mechanism of the formation of ultrathin gold nanowires [64].

**Figure 2:**
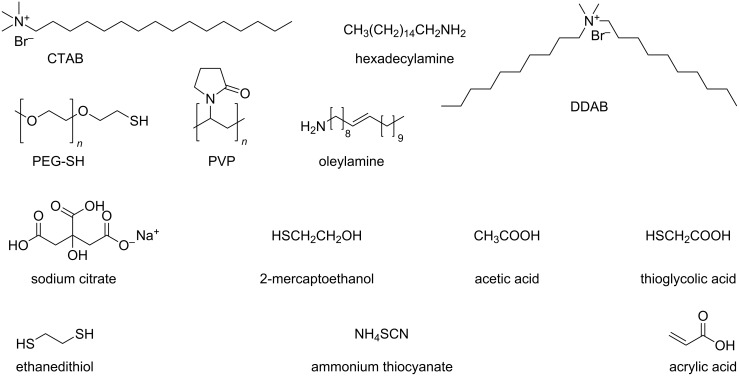
Common surface ligands for solution metal particle synthesis and ligand exchange. They provide colloidal stability to gold, silver, and copper nanostructures.

Silver nanoparticle synthesis by reduction of silver salts is an important case of ligand-directed nanoparticle growth. Cetyltrimethylammonium bromide (CTAB, Figure 2) in combination with ascorbic acid and spherical silver seeds yielded rod-like structures in water by reduction of AgNO_3_. Jana, Gearheart, and Murphy suggested that CTAB forms micellar templates for the anisotropic growth of nanostructures [65]. Recent studies modified this picture and indicated that CTAB passivates certain crystal faces (circumferential {100} planes), promoting the growth of nanorods along the ends. It depends on the structure of the initially formed seeds whether the growth occurs in one or two directions [66–67]. Silver nanobars were synthesized in ethylene glycol by the reduction of silver nitrate in the presence of poly(vinylpyrrolidone) (PVP, Figure 2) and sodium bromide. The nanobars formed when bromide ions etched the multiply twinned seeds, promoted the formation of single crystal seeds, and initiated anisotropic growth [68]. Longer silver nanowires grew from multiply twinned nanoparticles by the reduction of silver nitrate in the presence of PVP. The twin boundaries served as active sites for the addition of silver atoms as the strong interaction between PVP and the sides of the initially formed nanorod allowed preferential diffusion of the silver atoms to the ends of the nanorods, which grew into micrometer-long uniform silver nanowires [20,69].

Ligands also affect the geometry of gold and copper nanostructures that form in liquid. Cetyltrimethylammonium bromide and sodium oleate in an aqueous reaction mixture with hydrogen tetrachloroaurate trihydrate as precursor led to the formation of gold nanorods with controllable aspect ratios. In the presence of CTAB alone, rods with a broad size distribution formed (see above [65]) in a mixture with other shapes. An improved protocol with a binary surfactant mixture of CTAB and sodium oleate led to gold nanorods with a narrower size distribution (full width at half maximum between 110 and 170 nm) and shape impurities of less than 0.5% of the total number of nanoparticles. This uniformity was attributed to the coexistence of CTAB and sodium oleate; the oleate appears to mediate the binding between CTAB and certain facets of the growing nanorods [70].

Single crystalline gold nanoplates were prepared in water from hydrogen tetrachloroaurate and PVP using sodium citrate as reducing agent. The authors believe that low sodium citrate concentrations create small numbers of nuclei and that the remaining gold ions attach laterally on the nuclei so that triangular and hexagonal nanoplates with narrow size distribution form. Width and thicknesses of the nanoplates were controlled by changing the PVP concentration: An increasing PVP concentration decreased the number of nuclei and resulted in larger plates, while the absence of PVP led to irregular shapes (coin-like or rounded nanoplates) [71]. Wang et al. modified gold nanoparticle seeds (20 nm in diameter) with different bifunctional ligands and used them to initiate gold shell growth in a solution of HAuCl_4_, cetyltrimethylammonium chloride, and ascorbic acid. The morphologies of the resulting particles depended on the ligand; mercaptobenzoic acid (COOH) resulted in quasi-spherical particles, 4-aminothiophenol (NH_2_) led to spheres with multiple satellites, and 1,4-benzenedithiol (SH) yielded semi-shell or full-shell nanostructures with an internal gap between seed core and shell. The authors suggest that growth was controlled by the different binding affinities of the ligands to the gold atoms (COOH < NH_2_ < SH) that changed the interfacial energy between core and shell, resulting in different crystal growth modes [72].

Jin et al. controlled the shape of copper nanocrystals using hexadecylamine (Figure 2) as ligand and glucose as reducing agent. The amine showed good selectivity towards the {100} facets and prevented multiply twinned seeds from being etched, leading to copper nanowires by the reduction of CuCl_2_ in an aqueous dispersion. At lower amine concentrations, twinned seeds were etched and cube-shaped nanostructures predominated [73]. Didecyldimethylammonium bromide (DDAB) (Figure 2) in a mixture of Cu(acac)_2_ with phenyl ether and oleylamine (Figure 2) that normally yields copper nanospheres led to the formation of copper nanorods. Apparently, DDAB stabilized the {111} facets of the copper seeds and favored the crystal growth of the high-surface-energy ends of the rods [21]. Table 1 summarizes important ligands that have been used to direct the shape of metal nanoparticles.

**Table 1:** Ligand-directed shapes of coinage metal nanoparticles.

ligand	shape	metal [ref]

CTAB	rods	Ag [65]/Au [70]
PVP	bars/wires/rods	Ag [68–69,74]
PVP	plates	Au [71]
4-aminothiophenol	cores with satellites	Au [72]
1,4-benzenedithiol	semi shell/full shell	Au [72]
PEG-SH	rods	Au [75]
DDAB	rods	Cu [21]
hexadecylamine	wires/cubes	Cu [73]

The geometry of metal nanostructures can affect the percolation threshold and, thus, the conductivity of particle layers. For example, inks based on PVP-coated silver nanoparticles of different geometries were used to prepare conductive layers. Inks that contained only spheres (64 nm in diameter) formed layers with a conductivity of 769.2 S/cm at 70% metal mass fraction. Mixtures of spheres and plates (600 nm in length and 30 nm in thickness) or spheres and rods (5 μm in length and 100 nm in diameter) led to layers with conductivities of 14706 and 31250 S/cm already at 62 and 54% metal mass fraction, respectively [74]. Similarly, gold nanorods in composites with PEDOT:PSS yielded films that had higher conductivities than films filled with spheres. Both rods and spheres were capped with PEG-SH (Figure 2), a non-conductive ligand that was chosen because ethylene glycol (EG) is miscible with aqueous PEDOT:PSS solutions and the addition of EG to pure PEDOT:PSS films increases the electrical conductivity of the films. The packing of the rods and spheres in the films dominated their electrical characteristics, with rods percolating at lower filling ratios than spheres [75]. Even lower are the expected percolation thresholds for silver nanowires with high aspect ratios. They were applied as transparent, flexible conductors in many publications [76–79]. For example, silver nanowires capped by PVP provided layers of high transparency (90% transmission of the visible spectrum), excellent homogeneity, and low sheet resistance (*R*_s_ = 25 Ω/sq) in flexible electrodes [80].

### 2 Ligands in colloidal dispersions

The stability of colloidal dispersions depends on the capping ligands and affects shelf life and performance [32]. Ligands can trigger or prevent the agglomeration of metal nanoparticles depending on the solvent [81–82]. Ligand exchange may be necessary after synthesis to enable formulations with solvents that are suitable for deposition [83]. It is also possible to replace insulating ligands from synthesis with conductive moieties in order to avoid later sintering steps [83–84].

#### 2.1 Colloidal stability

Metal particles have large Hamaker constants, are strongly attracting each other, and only remain stable in dispersion if ligands reduce their attractive interactions [85]. The lower Hamaker constant of the ligand shell, its entropic behavior, and its ω-functionalities can make nanoparticles colloidally stable in organic or aqueous environments. Ligands such as citric acid [86], CTAB [66,70], or oleylamine [87] are commonly used on metal particles and provide some colloidal stability; thicker polymer shells in good solvents generally lead to greater stability [88–89]. The ligands are anchored on the cores through metal–ligand coordination, hydrophobic adsorption or electrostatic attraction.

In one study, alkylamines of different length stabilized copper nanoparticles in solvents such as THF or toluene. Longer chains increased stability and reduced metal deposition on the reactor walls [28]. The free electrons of nitrogen weakly coordinate the copper surface of the particles and the aliphatic chains stabilize the particles sterically; steric repulsion was strong enough only for chains above twelve carbon atoms to prevent rapid coalescence of the nanoparticles [28]. Karg et al. prepared gold nanoparticles in aqueous solutions and transferred them to chloroform and other solvents using amphiphilic alkylamines of different chain length to create organic dispersions of alkyl-stabilized nanoparticles [90]. Concentrated organic or aqueous dispersions enable particle printing using inkjet technologies, for example; exemplarily, field-effect transistors were inkjet-printed from inks based on silver–copper nanoparticles in 2-butoxyethanol acetate or electrocircuits from silver nanoparticles in water were inkjet-printed on paper substrates [50,91].

Alkanethiol-functionalized gold and silver nanoparticles in water exhibited increased dispersion stability with increasing length of the alkanethiol chain. Shorter alkanethiols (C2–C8) could not prevent aggregation and sedimentation of the nanoparticles; longer thiols (C12 & C14) resulted in higher colloidal stability. The effect was interpreted using an extended Derjaguin–Landau–Verwey–Overbeek (DLVO) theory that includes elastic repulsion and osmotic pressure as stabilizing mechanisms [92–93].

Polymers as capping agents typically form a thicker shell. Polyvinylpyrrolidone (PVP) in ethylene glycol [88] and polymethacrylic acid with thioether functionality in aqueous media [94] were introduced during synthesis in order to stabilize gold nanoparticles. The polymeric ligands created a hydrophilic coating around the gold nuclei that was permeable for tetrachloroaurate ions and allowed further grow while preventing aggregation. The polymeric chains provided steric repulsion and additional Coulomb repulsion in the case of polymethacrylic acid due to its potential to carry negative charge [94]. Que et al. reported that the size of the polystyrene block affected the stability of gold nanoparticles coated with a poly(ethylene glycol)-*b*-polystyrene diblock copolymer in water. A shorter polystyrene chain length led to a more compact coverage of the metal surface, rendering the gold nanoparticles more resistant and stable against aggregation in an electrolyte [95].

#### 2.2 Ligand-initiated agglomeration

Changes in the capping layer can induce agglomeration of dispersed nanoparticles or establish physical contact between the cores in deposited layers [96–97]. Silver nanoparticles stabilized by oleylamine ligands were immersed in 99.9% acetic acid in order to partially remove oleylamine, substitute it with acetic acid (Figure 2), and reduce the overall density of the ligand shell on the metal surface. The decrease in oleylamine coverage decreased the temperature required to establish physical contact between particles in dry films. Silver nanoparticles with reduced ligand coverage began to connect at approximately 100 °C and formed films with electrical properties that were superior to films of fully oleylamine-coated nanoparticles annealed at 200 °C [98]. In a different study from the same group, the authors prepared two inks of oleylamine-stabilized silver nanoparticles with core sizes of 5 and 12 nm. Dipping the printed nanoparticle films in methanol reduced the total weight of the oleylamine ligands by 7%. The reduced surface coverage facilitated desorption of the surface molecules and bridging of the metal cores at reduced temperatures [99].

Some ligands, for example 2-mercaptoethanol or thioglycolic acid, have been reported to cause chain-like agglomeration of gold nanoparticles during their deposition from an aqueous solution. In one study, the initially adsorbed sodium citrate (Figure 2) was replaced by 2-mercaptoethanol (Figure 2) in solution. This decreased the electrostatic repulsion between the nanoparticles and led to discrete, bifurcated or looped chains in suspension that were transferred to various solid substrates [100]. In a similar study, the exchange of citrate ligands by thioglycolic acid (Figure 2) allowed the authors to reduce the surface charge density, manipulate Coulomb repulsion between the particles, and initiate agglomeration in solution. Appropriate particle charges led to chain-like aggregates of the NPs as visualized by TEM imaging [101].

#### 2.3 Ligand exchange after synthesis

**Figure 3:**
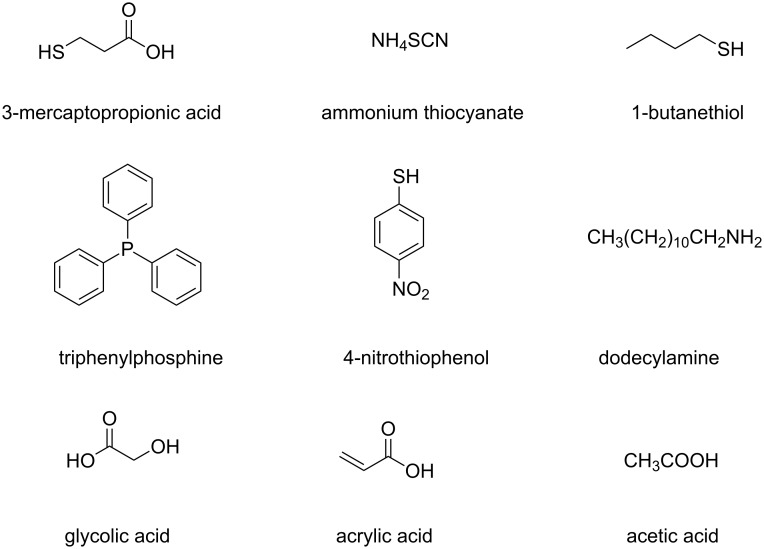
Representative examples of surface ligands that have been shown to improve charge transport properties in films.

Ligand exchange has been used to replace insulating ligands like oleylamine on gold nanoparticles with smaller ethanedithiol or ammonium thiocyanate molecules (Figure 2). Films of the modified nanoparticles exhibited resistivities that were six and ten orders of magnitude below that of films prepared from the original oleylamine-stabilized Au NPs [102]. The commonly used CTAB forms insulating layers, too, and has limited solubility even in water that makes ink formulation challenging. Exchanging CTAB with conjugated, thiophene-based polymers provided a stable ink that was used to deposit lines with good electrical conductivities [83]. Silver nanoparticles initially carrying oleylamine were coated with acrylic acid (Figure 2) that provided excellent stability in acetone or ethanol over a period of six months. Its low boiling temperature and molecular weight reduced the amounts of carbonaceous residues after annealing and led to continuous conductive layers at low annealing temperature (175 °C) [103]. Other researchers replaced oleylamine with acetic acid (Figure 3) as described in [98] (section 2.2) to create stable, ethanol-based Ag nanoparticle inks; they deposited layers that could be cured at 100 °C.

Exchanging hexadecylamine on the surface of copper nanowires (CuNWs) by polyvinylpyrrolidone (PVP) resulted in well-dispersed CuNW inks. The PVP chains stabilized the nanowires mainly via steric repulsion and chains of higher molecular weight endowed better colloidal stability according to turbidimetric measurements monitoring the change in backscattering intensity of light entering the NW dispersion. The conductivities of the CuNW films after sintering did not depend on the polymer chain length. Subjecting the sintered films to bending tests did not lead to a significant conductivity decrease. Embedding the NWs into a polymer provided mechanical flexibility without a loss of conductivity [104].

### 3 Ligands in particle films

Voluminous and dense ligand layers keep nanoparticle inks colloidally stable, but they may prevent electrical contacts in deposited films [32]. Charge transport can be improved after deposition by removing the surface-bound ligands [105], by linking nanoparticles chemically [106], or by ligand exchange in order to manipulate spacing in metal nanoparticle assemblies [107]. The following sections discuss the effect of ligands in the dry film and processes to modify them. Table 2 provides an overview of the correlation between coinage metal, ligand, deposition method, and film conductivity.

**Table 2:** Particle synthesis routes and deposition techniques reported for the preparation of coinage metal nanoparticles and their conductive films.

metal [ref]	shape	ligand	preparation	deposition	conductivity (S/cm)

Ag [74]	spheres	PVP	polyol	inkjet	769.2
Ag [74]	spheres + plates	PVP	chemical reduction	inkjet	14706
Ag [74]	spheres + rods	PVP	chemical reduction	inkjet	31250
Au [111]	spheres	ω-(3-thienyl)-hexanethiol	Brust [108]	spin-coating	2 × 10^−5^
Ag [112]	spheres	3-mercaptopropionic acid	chemical reduction	drying	980.9
Ag [30]	spheres	ammonium thiocyanate	microwave	spin-casting	1.13 × 10^5^
Au [33]	spheres	6-mercaptohexanoic acid + 3-mercaptopropanol	modified Brust	drying	2 × 10^5^
Au [114]	spheres	triphenylphosphine	interfacial deposition	interfacial deposition	30
Au [114]	spheres	4-nitrothiophenol	ligand exchange	immersion	750
Au [117]	spheres	decanethiol + 1,10-decanedithiol	two-phase method	substrate immersion	3.12 × 10^4^
Au [117]	spheres	decanethiol + 1,3-propanedithiol	two-phase method	substrate immersion	289 × 10^4^
Au [118]	spheres	dodecylamine + 1,9-nonadithiol	two-phase method	spin-coating	4.2 × 10^−3^
Au [120]	spheres	5-diphenylphosphino-2,2':5',2''-terthiophene	two-phase method	electrodeposition	3 × 10^−2^
Au [121]	spheres	tetraoctylammonium bromide + pyrrole or thiophene-based thiols	Brust [108]	immersion	10^−7^ to 10^−2^
Au [123]	spheres	1-butanethiol	phase transfer	spin-casting	1497
Au [124]	spheres	oleylamine	chemical reduction	drop-casting	3.1 × 10^5^
Ag [124]	spheres	oleylamine	chemical reduction	drop-casting	3.3 × 10^5^
Cu [46]	spheres	lactic acid	chemical reduction	paste depostion-drying	47.6
Cu [46]	spheres	glycolic acid	chemical reduction	paste depostion-drying	39.2
Ag [126]	spheres	dodecylamine	one-phase method	spin-coating	1205
Au [128]	spheres	Na_4_Sn_2_S_6_	ligand exchange	immersion	1000
Au [31]	spheres	citrate	Frens [86]	convective self-assembly	4545
Au [31]	spheres	bis(*p*-sulfonatophenyl) phenylphosphine dihydrate dipotassium	ligand exchange	convective self-assembly	0.333
Au [31]	spheres	mercaptopropionic acid	ligand exchange	convective self-assembly	0.625
Au [132]	spheres	citrate	Frens [86]	horizontal incubation	2000
Au [133]	spheres	thiophene-based terthiophene	two-phase method	drop-casting	4.8 × 10^−8^
Ag [134]	spheres	PVA	reduction by heating	drop-casting	225
Au [75]	rods	PEG-thiol	seed-mediated growth	drop-casting	2000
Ag [137]	spheres	dicarboxylic acid	combustion chemical vapor condensation	doctor blade	2 × 10^5^
Ag [142]	spheres	PEDOT:PSS	laser ablation	spin-coating	176
Au [83]	rods	PEDOT:PSS	ligand exchange	through masks	1428
Cu [143]	spheres	polypyrrole	chemical reduction	dry powder	0.208

#### 3.1 Conductivity

Electrical transport is sensitive towards the barrier created by the surrounding dielectric ligand shell of nanoparticles. Depending on the thickness of the barrier, electron transport has been described as quantum tunneling, electron hopping, ohmic conduction, or space-charge-limited conduction, to mention only a few [53,57,109–110]. Several reports stress that the thickness of the ligand shell strongly affects conductivity. Films of gold nanoparticles functionalized by thiophene-terminated dodecanethiols had 256-times lower conductivities than particles protected with thiophene-terminated hexanethiols. The decreased number of methylene units in the alkyl part of the thiols was deemed responsible for a decreased interparticle distance that improved film conductivity (to 2 × 10^−5^ S/cm) by electron hopping [111].

**Figure 4:**
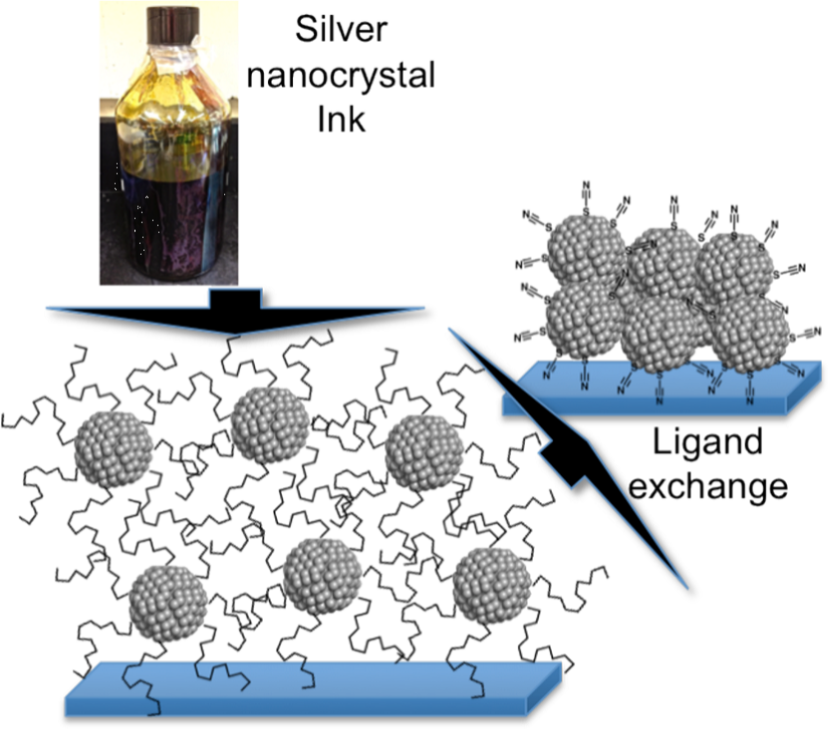
Post-deposition liquid exchange increases conductivity by replacing the insulating oleylamine ligand shell with ammonium thiocyanate. Reprinted with permission from [30], copyright 2014 American Chemical Society.

Films of silver nanoparticles exhibited a similar trend. Thiocarboxylic acids having three (Figure 3, 3-mercaptopropionic acid) and eleven carbon atoms in the alkyl chain were used as capping agents. Sheets of nanoparticles with short chains showed a conductivity (980.9 S/cm) that was ascribed to the shorter chain length and the lower grafting density, whereas sheets of particles functionalized with the longer thiocarboxylic acid were not conductive [112]. In a different study, exchanging oleylamine for the compact ammonium thiocyanate ligand (Figure 3) in the solid film (Figure 4) gave rise to highly conductive structures of silver nanoparticles (1.13 × 10^5^ S/cm) [30].

**Figure 5:**
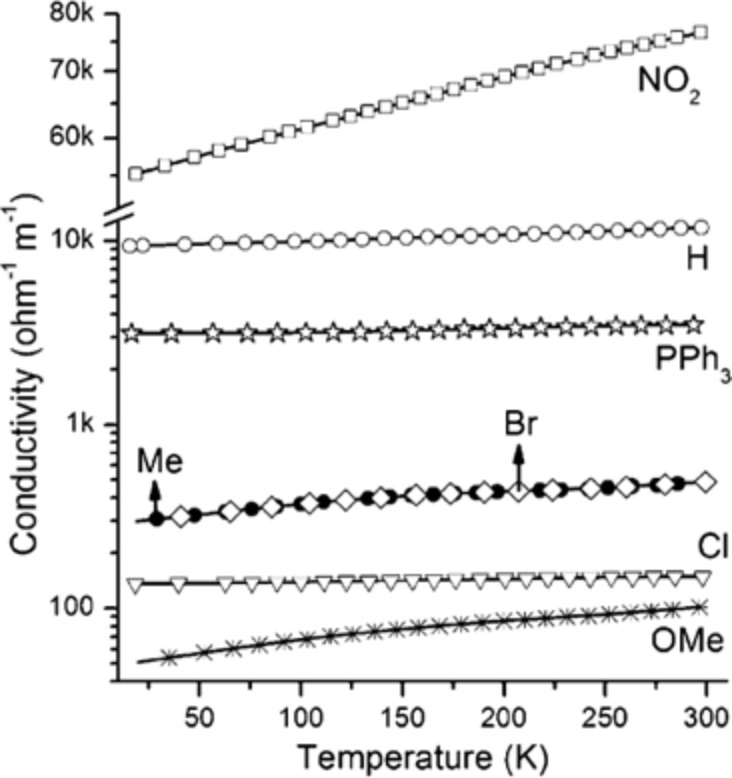
Conductivity as a function of the temperature in films of gold nanoparticles functionalized with thiophenol (H), with thiophenol *para*-substituted with nitro (NO_2_), methyl (Me), bromine (Br), chloride (Cl), or methoxy (MeO) groups, and with triphenylphosphine (PPh_3_). Reprinted with permission from [114], copyright 2012 American Chemical Society.

The packing density of ligands on the surface of metal nanostructures affects their dispersion stability [113] and the final film conductivity [112]. Films of silver nanoparticles with 3-mercaptopropionic acid as ligands were conductive, while the longer 11-mercaptoundecanoic acid yielded insulating films. The authors proposed that the longer ligands formed denser layers on the metal surface insulating the nanoparticles and rendering the films non-conductive [112]. The packing density of a ligand mixture can also influence the percolation transition temperature (*T*_p_), the temperature at which a metal nanoparticle film switches from an insulator to a conductor [33]. Elimination of a labile ligand (e.g., 3-mercaptopropanol) from the mixed ligand shell during purification of the gold nanoparticles in water created particles that were protected by only a sparse monolayer of ω-functionalized ionic ligands (25% surface coverage). The particles with the sparse layer remained well dispersed and had a lower *T*_p_ than those with a dense shell, so that conductive films (2 × 10^5^ S/cm) could be formed already at 145 °C. Charge transport also depended on the substituents in ligands, for example, in *para*-substituted thiophenol ligands on gold nanoparticles. Electron-donating substituents (Me, MeO, Cl, or Br) in *para*-position to the thiol group reduced the conductivity compared to films of triphenylphosphine (PPh_3_) (Figure 3) and thiophenol (H)-capped gold nanocrystals. The electron-withdrawing nitro group (NO_2_) caused an increase in conductivity from 30 S/cm (PPh_3_-capped Au NPs) to 750 S/cm as illustrated in Figure 5. The temperature profiles of the studied films all showed linear changes in conductivity that indicate a classical Arrhenius-type activated charge-carrier transport mechanism [114].

#### 3.2 Linking

**Figure 6:**
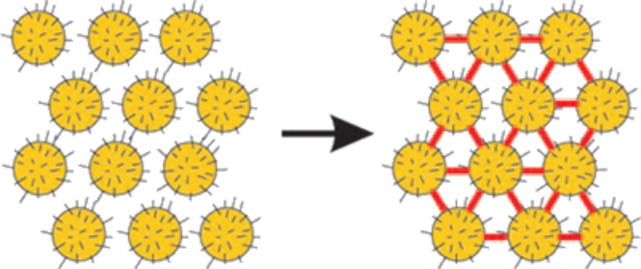
Cross-linking of gold nanoparticles stabilized by octanethiols and connected to each other via diarylethene molecules after the illumination with UV irradiation. Reprinted with permission from [116], copyright 2009 American Chemical Society.

Functional ligands that cross-link particles after their deposition as films can tune interparticle distances and conductivity [115]. Van der Molen et al. presented two-dimensional arrays of gold nanoparticles with octanethiol surface ligands that could be cross-linked by molecular insertion of diarylethene moieties. Hexagonally ordered particle layers were subjected to a ligand-exchange reaction and one single or a few connections between neighboring metal cores were established during illumination with UV light. The light-induced conductance enhancement of more than one order of magnitude suggested that the diarylethene molecular bridges created a percolating network (Figure 6) [116].

The length of linker molecules affects conductivity. An interdigitated microelectrode substrate was immersed into a mixture of decanethiol-functionalized gold nanoparticles and an alkyl dithiol (linking agent) to follow the formation of thin film assemblies. The conductivity of films prepared from nanoparticles of 2 nm in diameter and a dithiol having ten methylene groups was 3.12 × 10^4^ S/cm. A dithiol with only three methylene groups increased conductivity to 289 × 10^4^ S/cm [117]. In an another study, the dithiol 1,9-nonadithiol was spin-coated on a glass substrate followed by gold nanoparticles to create interconnected gold-nanoparticle assemblies with a homogeneous coverage and a film conductivity of 4.2 × 10^−3^ S/cm [118].

Cross-linking works for particles that are not prepared in liquids, too. A discontinuous film of gold was evaporated onto a mica substrate and exposed to the mobile oligomer 1,4-phenylene-diisocyanide (PDI). The molecule bridged the gold islands and increased conductivity from 6.5 × 10^−10^ to 270 × 10^−10^ S after 2000 s of exposure. The authors showed that the presence of two isocyanide groups in the PDI molecule is essential to achieve conductivity: Exposure to phenyl isocyanide hardly improved the conductivity [119].

Films of gold nanoparticles coated by conjugated phosphines were electrodeposited and coupled in situ through oxidation in a three-electrode electrochemical cell. The films of the cross-linked particles reached a conductivity of 3 × 10^−2^ S/cm, a value 1123-times higher compared to drop-casted films of unlinked particles with the same surface ligands. The authors suggest that the conjugated thiophene part of the phosphine ligands acted as a conductive pathway between the particles [120]. Similarly, alkyl-substituted pyrrole-, bithiophene-, or terthiophene-thiols were reacted with gold nanoparticles and oxidatively coupled gold particles in solution or in the solid state. Electron transport in the films was reported to reach conductivities in the range of 10^−7^ to 10^−2^ S/cm [121].

#### 3.3 Sintering

Instead of linking particles with organic molecules, one may sinter them to create metallic, conductive connections [32]. The conditions required for successful sintering depend on the tendency of the surface ligands to desorb, which lets particles fuse without melting [122]. Metal nanoparticle films with low insulator–metal transformation temperatures (*T*_p_) are desirable for applications on plastic films with low glass-transition temperatures (*T*_g_) [33,44]. The required annealing temperature is correlated with the length of the capping agents as reported by Gupta and co-workers. Inks of thiol-functionalized gold nanoparticles having thiols with eight, six, or four carbon atoms in their alkyl chains exhibited descending sintering temperatures from the longer thiol (204 °C) to the shorter (155 °C). The layer prepared from the C4-thiol-functionalized gold nanoparticles could be annealed at 160 °C, a temperature sufficiently low to create metallic films with a conductivity of 1497 S/cm on PET substrates [123]. A similar trend was observed in films of gold and silver nanoparticles stabilized by amines of different length. Films of metal nanoparticles stabilized by oleylamine (C_18_H_35_NH_2_) reached their maximum conductivity (3.1 × 10^5^ S/cm for Au NPs and 3.3 × 10^5^ S/cm for Ag NPs) at a temperature of 250 °C. Films of particles with dodecylamine (C_12_H_25_NH_2_, Figure 3) or octylamine (C_8_H_27_NH_2_) were already maximally conductive at 190–200 °C and 130–140 °C, respectively [124].

Silver-nanoparticle inks functionalized with carboxylic acids required sintering temperatures that depended on ligand chain length, too. Films of particles with C10-carboxylic acids heated to 140 °C did not become conductive. Layers printed from inks with a mixture of C10- and C6-carboxylic acids exhibited 29% of the conductivity of bulk silver after sintering at 140 °C. The authors suggest that the shorter acid first disassociated, which caused partial nanoparticle coalescence, released heat, and led to partial dissociation of the decanoic acid, and the formation of conductive patterns [125]. Copper nanoparticles with glycolic (Figure 3) and lactic acid ligands were also deposited as films. The authors report that the carboxylate moieties enabled ligand removal at low sintering temperatures (150 °C) so that nanoparticles coalesced and formed a continuous layer with conductivities between 39.2 and 47.6 S/cm [46].

The ω-functionalities of surface ligands affect the sintering process. In one example, the conductivity of films prepared from silver nanoparticles with dodecylamine began to rise significantly at 108 °C, whereas dodecylthiol led to an onset at 238 °C [126]. The weaker Ag–amine bond may explain the lower temperature threshold. Dodecylamine-functionalized Ag NPs were printed on polymers and reached a conductivity of 1205 S/cm after sintering at 140 °C [126].

Metal-chalcogenide complexes are a carbon-free alternative to stabilize nanocrystals in polar solvents such as dimethylformamide or methanol. Their use avoids undesired carbon species that create charge-trapping centers in the sintered films [127–128]. Capping gold nanoparticles with chalcogenides such as Na_4_SnS_4_, (NH_4_)_4_SnS_6_, or Na_3_AsS_3_ was facilitated by the nucleophilic nature of these ligands and the electrophilicity of insufficiently coordinated metal atoms at the crystal surface. The small chalcogenides supported the formation of close-packed nanoparticle solids, and their deposition at 80 °C led to efficient charge transport with enhanced conductivity values (10^3^ S/cm) compared to layers of dodecanethiol-capped gold nanocrystals (10^−9^ S/cm) [128].

In metal nanowires, materials that combine conductivity at lower percolation thresholds and mechanical flexibility, improved conductivity was achieved by means of conventional thermal annealing [129] or plasma treatment [130]. Gold nanowires (AuNW) with core diameters below 2 nm were arranged into a mesh on PET substrates using a nanoimprint technique and the insulating oleylamine ligand was removed by using a “soft” hydrogen/argon sintering process. The result was a transparent electrode that could be bent multiple times without losing more than one order of magnitude in conductivity [130]. Other reports describe the removal of capping ligands from AuNW using an ageing process. In a typical experiment, a freshly prepared dispersion of AuNW was left to stand at room temperature for 12 h and then drop-cast onto an air–water interface. The nanowires self-assembled into bundles, forming a continuous network. Transferring the mesh film onto PET substrates led to transparent electrodes that were flexible with a resistance increase of 7.2% upon substrate deformation [43]. A similar ageing process was used to create AuNW membranes. The dispersion was left to age for two days at room temperature, spread on the air–water interface, and transferred to a substrate by using a Langmuir–Blodgett trough. The single-layer nanomembrane that formed on the water surface had excellent optical transmittance of 90–97% and an electrical resistance of 1.14 × 10^6^ Ω/sq; it reacted to mechanical bending with a 14% increase in resistance after 400 bending cycles [131]. Note that there is no accepted standard yet for the measurement of the mechanical degradation in conductivity upon bending and the numbers provided can probably not be compared directly.

#### 3.4 Self-assembly

Ordered assemblies of metal nanoparticles provide defined film geometries that help investigating charge transport and the relationship between surface molecules and conductivity [53,107]. Self-assembly is commonly used to create such films. Moreira et al. reported on the role of different ligands such as citrate, phosphines, or thiols in the assembly of gold nanoparticles. Two different types of assemblies were obtained depending on the surface ligand. Gold nanoparticles stabilized by citrate assembled by forming connected islands of particles surrounded by intermittent voids. Despite the gaps a suppression of the tunnel height barrier and a core–core contact is suggested resulting in films with a conductivity of 4545 S/cm. Assemblies of phosphine- or thiol-functionalized nanoparticles created compact nanoparticle arrangements. Electron transport was dominated by the tunnel barrier imposed by the organic ligands. The conductivities of 0.333 and 0.625 S/cm obtained for the phosphine- or thiol-coated nanoparticles were consistent with the interparticle distances and their weak coupling [31].

In a different study, citrate-functionalized gold nanoparticles of two different sizes formed highly conductive films when the smaller nanoparticles filled the gaps between the larger ones. A film of the smaller nanoparticles was incubated in a solution of cysteamine and layering with alternating particle sizes created a uniform film. After five deposition cycles and smaller particles filling the interstices between the arrays of the larger ones, the film on glass reached a conductivity of 2000 S/cm [132]. Conventional drop casting of one size of gold nanoparticles functionalized by a π-conjugated terthiophene resulted in films of hexagonally packed particles. The terthiophene ligands aligned around the assembled particles, enabled face-to-face π–π interactions between the ligands, and resulted in a film conductivity of 4.8 × 10^−8^ S/cm. The low conductivity was attributed to the insulating alkyl chain between the terthiophene unit and the gold core [133].

**Figure 7:**
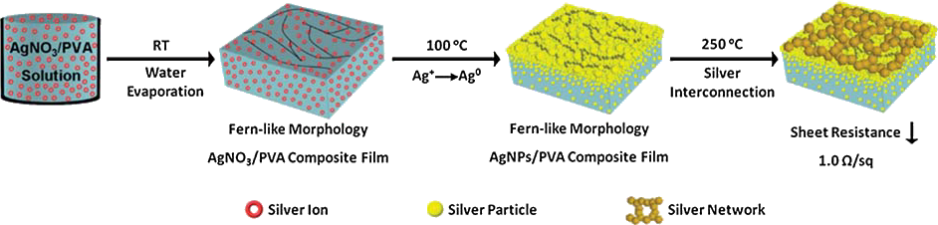
Silver-based transparent conductive films obtained through solvent evaporation, formation of the AgNO_3_/PVA hybrids with a fern-like morphology, and silver interconnection via annealing. Reprinted with permission from [134], copyright 2014 Royal Society of Chemistry.

An example of a polymer assisting the formation of assembled nanoparticles is given by Liang and co-workers. A solution containing AgNO_3_ and poly(vinyl alcohol) (PVA) was cast onto silicate glass. Evaporation of the solvent (water) led to the formation of a fern-like structure of PVA and AgNO_3_. A heat treatment reduced the silver precursor to metal structures that PVA stabilized in situ through its electron donating hydroxyl groups. The resulting film with the interconnected silver NPs (Figure 7) had a conductivity of 225 S/cm and was used to electrically connect light-emitting diodes [134].

### 4 Ligands in particle–polymer composites

#### 4.1 Formulations

Metal nanoparticles have been used to fill polymers and obtain conductive composites. When this is done from dispersion (rather than via melt compounding), ligands are required to keep the particles stable in the polymer solution [135]. For example, poly(ethylene glycol) thiol with an average number molecular weight of 1000 g/mol as capping agent kept spherical and rod-like gold nanoparticles dispersed in a formulation with PEDOT:PSS. Composite films with nanorod volume fractions between 10 and 70% were deposited onto quartz substrates; the film with the highest metal content reached a conductivity of 2000 S/cm without further processing, approximately twice that of neat PEDOT:PSS films [75]. Functionalizing silver nanoparticles with dicarboxylic acids (oxalic to octanedioic acid) [136] improved the dispersion stability and enabled increased loadings up to 80 wt % of silver nanoparticles and silver flakes in a non-conjugated epoxy resin. Sonicating the silver NPs with surfactants resulted in surfactant-coated particles and increased the composite conductivity to 2 × 10^5^ S/cm compared to untreated silver nanoparticles (5 × 10^−7^ S/cm). Sintering at 150 °C further improved conductivity [137].

Jiang et al. reported the effects of ligand length on the sintering behavior of silver nanoparticles in an epoxy-based composite. Particles with longer surface ligands limited the conductivity after sintering to 1.85 × 10^−5^ S/cm, while shorter ligands led to values up to 4166 S/cm [138].

#### 4.2 Particle–polymer bonds

**Figure 8:**
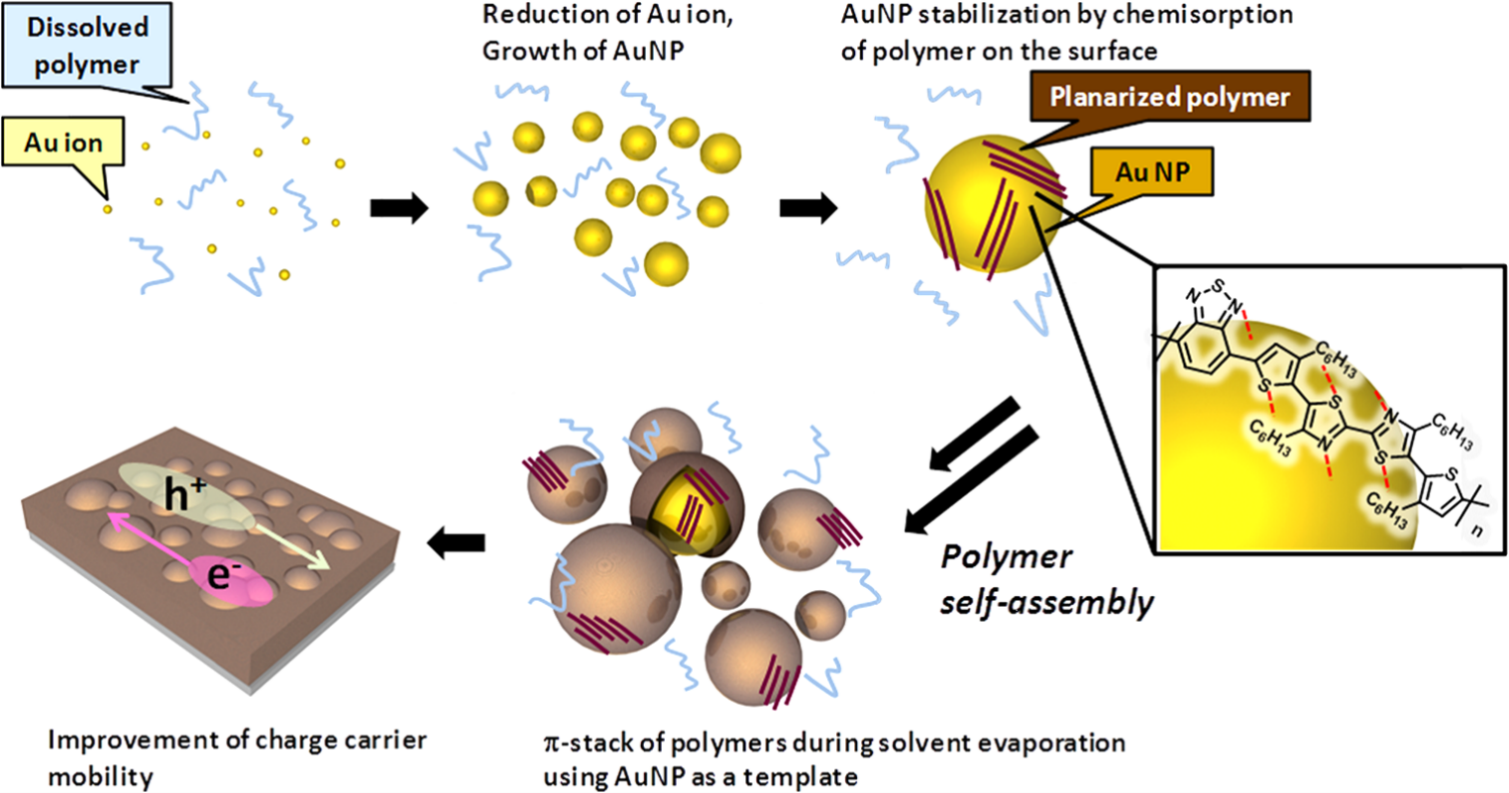
Formation of gold nanoparticles in a bithiazole–benzothiazole-based polymer matrix and their role as a template for the self-assembly of the bulk polymer leading to enhancement of the charge-carrier mobility. Reprinted with permission from [141], copyright 2012 American Chemical Society.

Polymers that are directly attached to the surface of metal nanoparticles lead to a different class of composites. Grafting or growing polymer chains from the metal surface can provide well-defined interfaces between polymer and particles that are favorable for charge transport [139–140]. Vijayakumar et al. stabilized gold nanoparticles of 10 nm average diameter with a conjugated copolymer comprising bithiazole and benzothiazole. The sulfur and nitrogen atoms in the blocks interacted with the metal surface, providing stability to the nanoparticles. The nanoparticles with the chemisorbed polymer acted as a template for the self-assembly of additional bulk polymer during solvent evaporation. Improved planarization and ordering of the bulk polymer during solidification enhanced the effective conjugation length and the delocalization of the π-electrons. Electron and hole mobilities of the solid composite with 5 wt % of gold were two to twelve times higher than in the unfilled polymer (Figure 8) [141].

Semaltianos et al. prepared ligand-free silver nanoparticles in deionized water by laser ablation. Their colloidal solution was mixed with the polymer mixture PEDOT:PSS, which coated the metal surface. The sulfur atom of the thiophene ring is shown to form a bond with the silver NPs and resulted in a uniform distribution of the nanoparticles in the composite. Films of the composite prepared by spin coating exhibited a 1.5-times higher electrical conductivity (176 S/cm) compared to films prepared only from the PEDOT:PSS mixture (125 S/cm) [142].

Other researchers employed ligand exchange to attach PEDOT:PSS on the surface of gold nanorods that have lower percolation thresholds than spheres of equivalent volume. The original surface ligand used in nanorod synthesis (CTAB) forms insulating barriers in films. A thin PEDOT:PSS layer on the surface of the nanorods replaced the CTAB, provided stable dispersions in different solvents, and allowed for the preparation of conductive films with comparatively high conductivities (1428 S/cm) without sintering, comparable to that of conventional metal-nanoparticle inks after sintering [83]. Copper nanoparticles in a polypyrrole matrix altered the morphology of the unfilled polymer and changed electrical conductivities from 0.012 S/cm to 0.208 S/cm. Annealing increased the conductivities of both materials; the authors suggest alignment of the polymeric chains and an increase of the effective correlation length as mechanism [143].

## Conclusion

Metallic nanoparticles can be wet-deposited on various substrates by using a wide range of coating and printing techniques in order to obtain conductive tracks. Among the coinage metals, silver nanoparticles are most commonly used in inks due to their high conductivity and relative stability. Gold is commonly applied if improved stability or biocompatibility is required. The synthesis and formulation of metal nanoparticles as inks requires appropriate ligands. Current literature provides many examples of ligands that provide various functions beyond limiting particle size during synthesis and colloidal stability during formulation. Small inorganic ligands such as chalcogenides, conductive polymers and metallic coatings, for example a silver shell, yield inks with different interesting properties. They help to pack the conductive nanoparticles more closely or provide sintering-free inks, for example.

The price of commercially available gold and silver-based inks limits their industrial use in some applications, and research into low-cost alternatives such as copper is underway. Progress is required to counter the inherent tendency of copper nanoparticles to oxidize and loose stability in the inks; a dense ligand layer for example based on polymers may be a viable strategy, but there exists a tradeoff between the gain in stability and retaining high film conductivity at minimum sintering temperatures. Conjugated, conductive polymers as capping agents are an emerging option that could yield hybrid, stable particles with low contact resistances at well-defined inorganic-organic interfaces.

The advent of the bulk solution synthesis of silver nanowires brought new dynamics to the field of nanoparticle-based transparent materials. Polyol synthesis of Ag NWs with PVP as shape-directing agent is scalable, but current methods provide very limited control over the wire geometry; currently available Ag NWs cause haze and coloration. In the future, curtailed ligands should provide better definition of the particle size and concurrently improve junction resistance. The same is true for other anisotropic particles that have a potential for electronics: Oleylamine-coated gold nanowires, for example, are too instable to directly use them as inks today, but use of appropriate solvents could alleviate this limitation. This dynamic field at the border of chemistry and materials science will continue to grow.
